# Effectiveness of a positive psychology and mindfulness-based app on mental health for parents of children with a neurodevelopmental disorder: study protocol of a pragmatic international randomized controlled trial

**DOI:** 10.1186/s13063-024-08256-w

**Published:** 2024-06-26

**Authors:** Kim J. M. Tönis, Constance H. C. Drossaert, Peter M. ten Klooster, Marie Schaer, Thomas Bourgeron, Jan K. Buitelaar, Yair Sadaka, Christine M. Freitag, Keren Mayer Lapidus, Andreas G. Chiocchetti, Wouter G. Staal, Ernst T. Bohlmeijer

**Affiliations:** 1 Faculty of Behavioural, Management and Social Sciences, Section of Psychology, Health and Technology, University of Twente, 217 Enschede, The Netherlands; 2 Department of Psychiatry, Faculty of Medicine, University of Geneva, Geneva, Switzerland; 3 Human Genetics and Cognitive Functions, Institut Pasteur, UMR3571 CNRS, IUF, Université Paris Cité, Paris, France; 4 Pediatric Neurology Unit, Pediatric Division, Soroka Medical Center of the Negev, Beer Sheva, Israel; 5 Department of Child and Adolescent Psychiatry, Goethe University, Frankfurt Am Main, Germany; 6 Negev Child Development Center of the Israel Ministry of Health, Beer Sheva, Israel; 7 Donders Institute for Brain, Cognition and Behavior, Radboud University Medical Centre, Nijmegen, The Netherlands; 8 Institute for Brain and Cognition, Leiden University, Leiden, The Netherlands; 9 Karakter Child and Adolescent Psychiatry University Centre, Nijmegen, The Netherlands

**Keywords:** Randomized controlled trial, RCT, Effectiveness, Application, Self-help, Digital intervention, Parents, Neurodevelopmental disorder, Autism, ADHD, Well-being, Ability to adapt, Adappt

## Abstract

**Introduction:**

Parents of children with a neurodevelopmental disorder (NDD) experience more stress than parents of typically developing children. In a cocreation process with experts and parents, a low-threshold application that uses exercises based on the principles of positive psychology and mindfulness was developed. This application, called “Adappt,” aims at enhancing the ability to adapt of the parents and caregivers of children with NDDs and at supporting their mental health. This protocol describes the evaluation study of the effectiveness of Adappt, its core working mechanisms and user experiences.

**Method:**

A pragmatic international multicenter randomized controlled trial will compare the effectiveness of Adappt with a (delayed) waitlist control condition. At least 212 parents or primary caregivers of children younger than 18 years diagnosed with or suspected of a NDD will be randomly assigned to the intervention or waitlist control condition. Participants are excluded if they have severe anxiety or depression levels or are in treatment for mental health issues. Measures will be collected online at baseline, post-intervention (1 month after baseline), and 4 and 7 months after baseline. The primary outcome is the improvement in generic sense of ability to adapt as measured with the Generic Sense of Ability to Adapt Scale (GSAAS; (Front Psychol 14:985408, 2023)) at 4-month follow-up. Secondary outcomes are mental well-being, (parental) distress, and client satisfaction with “Adappt.”

**Discussion:**

Results of this study will contribute to knowledge on the effectiveness of a low-threshold application for parents of children with a NDD in multiple countries. If the application is found to be effective in improving mental health, recommendations will be made for implementation in health care.

**Trial registration:**

This study is registered on clinicaltrials.gov (NCT06248762) on February 8, 2024, and the Open Science Framework (https://osf.io/5znqv).

**Supplementary Information:**

The online version contains supplementary material available at 10.1186/s13063-024-08256-w.

## Introduction

The concept of neurodevelopmental disorders (NDDs) is based on criteria defined in the classification system of Diagnostic and Statistical Manual of Mental Disorders, Fifth edition (DSM-5) of the American Psychiatric Association. Whether NDDs are understood as disorder, disability, or just a difference between people depends on both context and culture. Where a health-care perspective is taken, the focus is on the impairments people with NDDs experience. NDDs are thought to be caused by an early disruption of the brain development with a negative impact on behavior, cognition, motor control, self-help and social communication [67]. Attention deficit hyperactivity disorder (ADHD), autism spectrum disorder (ASD), communication disorders (CDs), motor disorders (MDs) including tic disorders, intellectual disabilities (ID) and specific learning disabilities (SLDs) are classified under NDDs in the DSM-5TR [1]. Francés et al. [31] performed a systematic review on the prevalence of NDDs in children and found the prevalence rates ranging between 5 and 11% for ADHD, 0.7 and 3% for ASD, 1 and 3.4% for CDs, 0.8 and 17% for MDs, 0.6% for ID, and 3 and 10% for SLD.

NDDs not only affect the life of the persons or children with this condition, but may also have a profound impact on their families. Parents of children with ASD [9, 38], ADHD [71], or NDDs [18] have shown to experience more stress than parents of children with a typical development. A large body of research has been conducted on (causes of) stress in parents of children with NDDs, mainly in parents of children with ASD [24]. For example, NDDs are associated with a higher prevalence of sleep issues impacting the well-being of the family [5]. In addition, a significant association was found between parenting self-efficacy and well-being in mothers of children with ASD [51]. Furthermore, difficulties with understanding or dealing with the child’s behavior, struggles with support and care, and social stigma contribute to parental stress [9]. Taking care of a child with a NDD can also be challenging because the child’s needs change over time [10] and parents continuously face new challenges [22]. Therefore, being able to adapt to new challenges and changing situations is crucial for the mental health of these parents. Recently, a self-help application has shown to significantly improve the perceived ability to adapt in people with mild to moderate distress [73]. This shows the potential of improving the ability to adapt through digital interventions.

Although (early) interventions targeted at the child’s behavior or development can improve adaptive behavior of children with ASD [76] and ADHD symptoms in children with ADHD [16], these interventions did not have a significant effect on parenting stress [16]. Increased levels of parental stress are associated with a reduced family life dimension of parental quality of life [30], parental quality of life [56], health-related quality of life of children [15], and negatively influence the ability to raise the child (i.e., care and behavior management) [9]. This highlights the need to actively support the mental health of parents of children with a NDD.

A wide range of interventions have been developed to improve mental health of parents of children with NDDs, including mindfulness and positive psychology interventions (PPIs) [20, 22, 33, 53]. For instance, research suggests that parenting stress, anxiety, and depression can be improved through a diversity of interventions (e.g., mindfulness and PPIs) in parents of children with ASD [22]. Mindfulness training has also shown to be effective in reducing parenting stress in parents of children with ADHD [53, 70] and mothers of children with ASD [25]. Mindfulness is defined as being fully aware of cognitions, emotions, and experiences with a non-judgmental attitude from moment-to-moment [7, 44], and is supposed to improve emotion regulation [69]. This is especially relevant for parents of children with NDDs, since disturbances in emotion regulation of parents were found to correlate significantly with parental stress in parents of children with ASD [43]. PPIs are interventions that aim to promote positive behavior, cognitions, and feelings [64]. PPIs can consist of single interventions focusing on specific aspects such as gratitude, optimism, or meaning making. Combinations of interventions are called multicomponent PPIs (mPPIs) [40]. A recently performed large meta-analysis (*n* ≥ 72,000 participants) showed the effectiveness of PPIs for both clinical and non-clinical samples on various indicators of mental health [13]. Interventions consisting of multiple PPIs tended to have larger effects on mental health than single PPIs, and longer PPIs resulted in larger effects on mental health [13].

Traditional face-to-face (group-) interventions can have disadvantages that can cause additional stress, especially for parents of children with a NDD, such as availability restrictions, waitlists, and issues with arranging a babysitter [57, 72]. Therefore, flexible, low-threshold, and low-invasive supportive digital interventions for parents of children with a NDD seem to be a promising avenue to support their mental health. Digital interventions have the advantage of being broadly available and accessible without waitlists. In addition, research has shown that both mindfulness and PPIs can be successfully digitally delivered. Gál et al. [34] performed a meta-analysis of RCTs on mindfulness interventions delivered as digital application and found significant effects on anxiety, depression, perceived stress, and psychological well-being in diverse populations (e.g., students, clinical samples, general population). Furthermore, El Menshawy [26] performed a meta-analysis of online delivered PPIs (oPPIs) and found small but significant effects on well-being and depressive symptoms for adults. This indicates that digital interventions based on mindfulness and positive psychology can be used to improve mental health.

However, evidence on the effectiveness of interventions to improve mental health in parents of children with NDDs is scarce. Da Paz and Wallander [22] performed a review of these interventions especially for parents of children with ASD and concluded that available trials rarely focused on the long-term effects (i.e., > 3 months), sample sizes were generally small and generalizability is limited due to homogeneous samples (e.g., mainly mothers aged around 40). This indicates that there is a need for more robust research on interventions for this target group to support more definitive and generalizable conclusions. Furthermore, insight in core working mechanisms underlying the (potential) effects of PPIs on mental health-related outcomes for this target group is lacking.

Recently, researchers of the University of Twente developed a supportive self-help application (app) based on positive psychology and mindfulness, called “Adappt”, in cocreation with experts and parents. This app consists of evidence-based exercises based on the principles of positive psychology and mindfulness. The primary aim of the current intervention study is to examine the effectiveness of this intervention on the ability to adapt of parents of children with a NDD in an international randomized controlled trial with waitlist control condition. The secondary objectives are to evaluate (1) its effects on mental health including mental well-being, perceived stress, anxiety, depression, (2) its effects on parental experiences including parental self-efficacy, positive aspects of caregiving, (3) the role of core working mechanisms of the intervention including mindfulness, savoring, self-reassuring, psychological flexibility, and positive coping skills on the ability to adapt and mental health-related outcomes, and (4) users’ acceptance of and satisfaction with the intervention.

## Method

The Standard Protocol Items: Recommendations for Interventional Trials [14] guideline was followed to report this protocol. The study protocol and analysis plan were preregistered on the Open Science Framework (OSF, https://osf.io/5znqv) and the clinical trials register clinicaltrials.gov (NCT06248762).

### Study design

A non-blinded pragmatic international randomized controlled trial will be performed to study the effectiveness of Adappt in parents of children with a NDD. Participants will be recruited in France, Germany, Israel, Switzerland, and The Netherlands. However, participants from other countries that are able to understand one of the available languages (French, German, Hebrew, and Dutch) can sign-up for the study and participate if they meet the eligibility criteria.

**Fig. 1:**
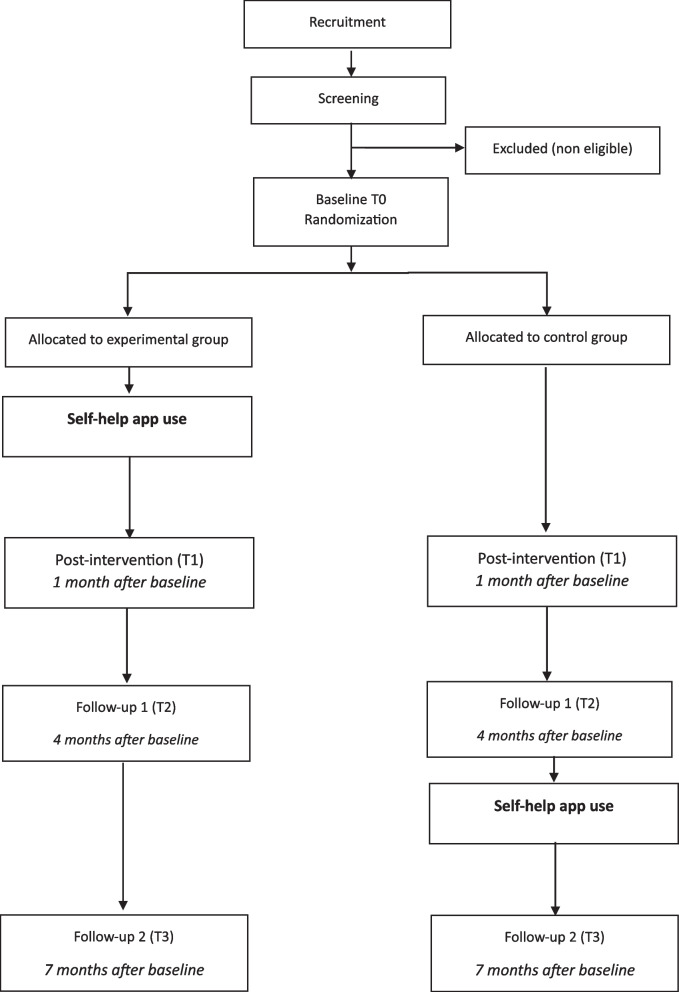
Flowchart of participants

**Fig. 2:**
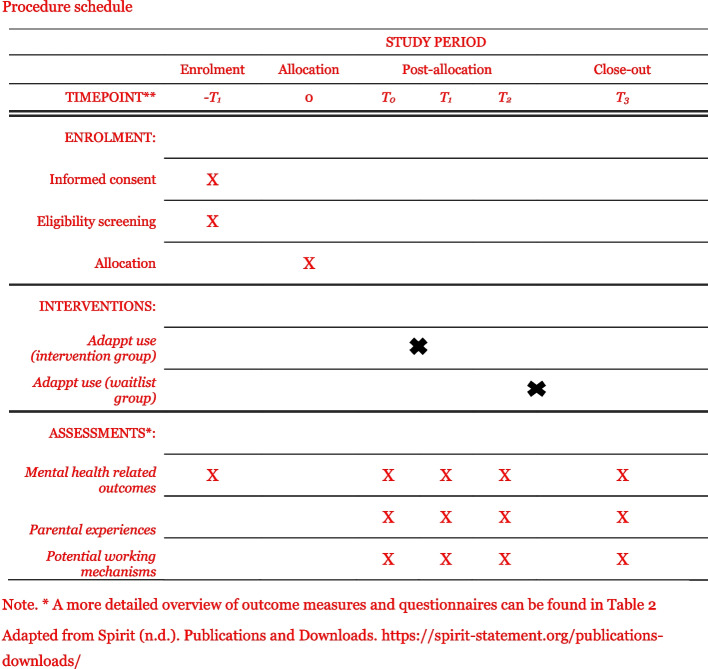
Procedure schedule. Note. * A more detailed overview of outcome measures and questionnaires can be found in Table 2. Adapted from Spirit (n.d.). Publications and Downloads. https://spirit-statement.org/publications-downloads/

After recruitment via service providers, parent associations, and social media (see “study procedure below”), interested parents fill in a screening form to check for eligibility. Eligible parents receive an e-mail with a link to the (online) baseline questionnaire (T0) and will then be randomized 1:1 to the experimental condition or waitlist control condition. Those randomized to the experimental condition start using the (unguided) digital intervention after the online baseline. One month after baseline participants will complete the post-measurement (T1). Furthermore, two follow-up measures will be conducted, at 4 months (T2) and 6 months after baseline (T3). Those randomized to the control condition receive the intervention that takes 1 month to complete after T2. The flow of participants and measurements can be found in Figs. 1 and 2.

### Participants

Eligible participants need to be (1) at least 18 years of age, (2) parent or primary caregiver of one or more children (younger than 18 years) diagnosed with or suspected of a NDD, (3) in possession of an e-mail address, (4) in possession of a smartphone or tablet with a stable internet connection that can be used to download and use the self-help app, and (5) willing to use the self-help app for a month, for approximately 10–15 min a day.

Since this self-help app intervention aims to support parents in reducing stress and promoting adaptive abilities, and is not a treatment for severe mental complaints, the following exclusion criteria are used: (1) being in treatment for a mental health issue, (2) presence of severe symptoms of anxiety as indicated by a score of at least 15 on the Generalized Anxiety Disorder (GAD-7; [66]), and (3) presence of moderately severe to severe symptoms of depression, as indicated by a score of at least 15 on the Patient Health Questionnaire (PHQ-9 [49],). Excluded participants will be informed about the reason for exclusion and those excluded for moderate to severe anxiety and/or depression levels will be advised to contact their local general practitioner.

### Study procedure

Participants will be recruited in the five countries (France, Germany, Israel, Switzerland, and The Netherlands), via (1) the social media channels of relevant stakeholders involved in the project, (2) associations for parents of children with NDDs, and (3) information leaflets and through health practitioners informing parents about the study in clinical centers for children with NDDs. Interested parents will be forwarded to a website of the study. At the website, the parent(s) will find more information about the study, the participant information letter, the option to ask questions via e-mail and have the option to register for the study. Therefore, participants will be referred to a Qualtrics questionnaire where they can register themselves and give informed consent, following the procedure of Kloos et al. [48]. Participants actively confirm that they (1) have read and understand the study information, (2) had time to consider participation and potential questions have been answered, (3) give consent for participation in the study, (4) know that participation is voluntary and withdrawal of consent can be done any time without giving a reason, and (5) consent to the use of their research data as described in the participant information letter.

Participants who give consent will be directed to the screening questionnaire, including questions related to in- and exclusion criteria (i.e., age of the participant, diagnosis, and age of the child with a NDD, being in possession of an e-mail address, smartphone/tablet, and stable internet connection, willingness to use the intervention, the PHQ-9, the GAD-7, and current treatment for a mental health issue). Recruitment will start in September 2024. It is expected that the required number of 212 participants (see “Sample size calculation”) will be reached in January 2025.

Eligible participants will receive a link to the online baseline questionnaire (T0) in Qualtrics, which takes about 30 min to complete. New T0-completers will be randomized once a week to the control or experimental condition and informed via e-mail about the allocation (see “Randomization and treatment allocation”). All participants allocated to the experimental condition will also receive the download instructions and can start using the intervention.

Parents will be invited to use the intervention daily for at least 30 days for 10–15 min a day. One month after baseline, all participants receive the post-intervention questionnaire (T1). The T1 measurement of the experimental condition will include additional questions to evaluate their satisfaction with and use of the intervention. It will take about 30 min (control condition) to 45 min (experimental condition) to complete T1. Four months after baseline, all participants receive the first follow-up questionnaire (T2), which takes about 30 min to complete. After the follow-up assessment control condition participants receive the download instructions and can use the intervention. Three months after the first follow-up questionnaire (7 months after baseline), all participants receive the second follow-up questionnaire (T3). The T2 of the control condition includes additional questions to evaluate the intervention and takes about 45 min to complete, whereas the questionnaire for the intervention condition takes about 30 min to complete. Participants that decided to withdraw from the study receive the opportunity to give feedback on the intervention and/or study.

### Randomization and treatment allocation

T0-completers will be randomly assigned to the experimental or waitlist condition by the principal investigator using central block randomization, with a block size of 4. A priori created randomization schemes will be made using www.sealedenvelope.com. This tool provides random lists that will be combined with the participant IDs to randomly allocate participants to the experimental condition or waitlist control condition with an allocation ratio of 1:1.

### Intervention

The Adappt intervention is based on positive psychology and mindfulness and was developed in cocreation with the target group and relevant key stakeholders including professionals working with children with a NDD and their parents (e.g., life-course supporters, social psychiatric nurse, school care coordinator, clinical psychiatrist and teacher at school for children with a NDD) in order to maximize fit of content and increase acceptance. The self-help app consists of a variety of positive psychology and some mindfulness-based exercises. The exercises are based on proven to be effective positive psychology interventions, and the intervention is structured around six themes with five exercises each (see Table 1). Participants will be guided through the intervention by receiving one exercise a day of a theme of choice. Several persuasive technology elements are included in the app to improve adherence to intervention [46], such as guiding participants through the application (tunnelling), and notifications to remind the participant of practicing. The app has been developed by LearnEnjoy (https://learnenjoy.com), a French company specialized in the development of digital interventions for people with and without a NDD.

**Table 1:** Overview of positive psychology themes

Theme	Description	Examples of exercises
Strength-based empowerment	Learning and applying your own strengths	Using strengths [58]
Points of light	Attention for the nice small things can be helpful	Three good things [63]
Attention for yourself	Taking care of yourself	Best possible self [61]
Improved connectedness	Improve relationships through attention and gratitude	Acts of kindness [21]
Accept & Let go	Change what you can change, accept what you cannot change and wisdom to see the difference	Cognitive defusion [3]
Asking & Getting help	Ask, refuse, and accept help	Gratitude diary [45]

### Waitlist control condition

Participants in the waitlist control group can start the self-help app after completing the first follow-up questionnaire, 4 months after baseline. The decision for a waitlist control has been made for ethical reasons, since these participants indicated a need for support by signing up for the intervention and we considered it not appropriate to withhold these participants from support for a longer period. With this design, the control group no longer serves as a control after the first follow-up questionnaire (4 months after baseline), since the participants may have then also started to use the self-help app.

### Outcome measures

All study measurements will take place online via the online survey program Qualtrics (https://www.qualtrics.com). An overview of the outcome measures and measurement points can be found in Table 2. Measures that were not available for a certain language were translated by native speakers with back-and-forth translation.

**Table 2:** Overview of outcome measures and measurement points

Questionnaire	Number of items	Outcome measure	Screening	T0 (baseline)	T1 (Post-intervention)	T2 (follow-up 1)	T3 (follow-up 2)
GSAAS	10	Ability to Adapt		X	X	X	X
MHC-SF	14	Mental well-being	X	X	X	X	X
PSS	10	Parenting stress		X	X	X	X
HADS-A	7	Anxiety symptoms	X	X	X	X	X
PHQ-9	9	Depressive symptoms	X	X	X	X	X
CAPES-DD	24	Parenting self-efficacy and behavior of the child		X	X	X	X
PAC	9	Positive aspects of caregiving		X	X	X	X
SBI	8	Savoring		X	X	X	X
FSCRS-SF	5	Self-reassuring		X	X	X	X
AAQ-II	7	Psychological flexibility		X	X	X	X
CERQ	16	Positive coping skills		X	X	X	X
MAAS	15	Mindfulness		X	X	X	X
Characteristics		Parental and child characteristics	X^d^	X^d^			
CSQ-8		Client satisfaction			EXP		WL

*EXP* Experimental condition, *WL* Waitlist condition

^a^5 weeks after baseline

^b^4 months after baseline

^c^7 months after baseline

^d^The majority of the characteristics are asked at T0; however, those relevant for inclusion are asked in the screenings questionnaire

#### Primary outcome measure

The primary outcome, ability to adapt will be measured with the 10-item Generic Sense of Ability to Adapt Scale (GSAAS; [32]). Items will be answered on a 5-point scale ranging from not at all (0) to always (4). An average score will be calculated, with a higher score being indicative of higher perceived ability to adapt. Psychometric properties of the GSAAS were found to be good [32].

#### Secondary outcome measures

##### Mental health-related outcomes

Mental well-being will be measured with the 14-item Mental Health Continuum – Short Form (MHC-SF; [47]) with three subscales: emotional (3 items), social (5 items), and psychological (6 items) well-being. Items will be scored on a 6-point scale ranging from never (0) to every day (5). Averages will be calculated for the total mental well-being scale and the three subscales, with higher scores being indicative for more mental, emotional, social, and psychological well-being. Psychometric properties were found to be good [52].

Perceived stress will be measured with the 10-item perceived stress scale (PSS; [17]). Items will be scored on a 5-point scale ranging from never (0) to very often (4). Sum scores will be calculated (0–40), with higher scores being indicative for larger levels of perceived stress. Psychometric properties have been found to be acceptable [54].

Anxiety symptoms will be measured with the 7-item Anxiety subscale of the Hospital Anxiety and Depression Scale (HADS-A; [77]). Items will be scored on a 4-point scale ranging from most of the time (3) to not at all (0). Sum scores will be calculated (0–21), that can be interpreted as no symptoms of anxiety (0–7), possibly an anxiety disorder (8–10), and likely an anxiety disorder (11–21) [42]. Psychometric properties were found to be good [6].

Depressive symptoms will be measured with the 9-item Patient Health Questionnaire (PHQ-9; [49]). A 4-point scale ranging from not at all (0) to nearly every day (3) will be used to score items. Sum scores will be calculated (0–27), that can be interpreted as mild (5–9), moderate (10–14), moderately severe (15–19), and severe (20–27). Psychometric properties were found to be good [49].

##### Parental experiences

Parental self-efficacy (16 items), behavioral (10 items), emotional (3 items), and total (16) problems of the child and prosocial behavior (8 items) of the child will be measured with the 24 item Child Adjustment and Parent Efficacy Scale – Developmental Disability (CAPES-DD; [27]). Parents rate their confidence in managing behavior of the child on a 10 point scale, ranging from “certain I cannot manage” (1) to “certain I can manage it” (10), resulting in a sum score between 16 and 160, with higher scores being indicative of higher parental self-efficacy levels. Child behavior items will be scored on a 4-point scale from not at all (0) to very much (3). Sum scores will be calculated for the subscales emotional problems (0–9), behavioral problems (0–30), prosocial behavior (0–24), and total problems (0–48). Psychometric properties were found to be very good [27].

Positive aspects of caregiving will be measured with the 9-item Positive Aspects of Caregiving (PAC; [68]). Items will be scored on a 5-point scale ranging from disagree a lot (1) to agree a lot (5). A sum score will be calculated (9–45), with higher scores being indicative of more positive aspects of caregiving. Psychometric properties were evaluated and found to be good [68].

##### Potential working mechanisms

Savoring will be measured with the 8-item savoring the moment subscale of the Savoring Beliefs Inventory (SBI; [12]). A 7-point scale from strongly agree (1) to strongly disagree (7) will be used to score items. Since four items are positively formulated and four are negatively formulated, sums score ranges from − 24 to + 24, with higher scores being indicative of a stronger savoring in the moment. Psychometric properties were found to be good [12, 37].

Self-reassuring will be measured with the 5-item reassuring self-subscale of the Forms of Criticism/Self-Attacking and Self-Reassuring Scale Short Form (FSCRS-SF; [36, 65]). Items will be scored on a 5-point scale ranging from not at all like me (0) to extremely like me (4). Sum scores will be calculated (0–20), with higher scores being indicative for a larger sense of self-reassurance. Psychometric properties were evaluated and found to be good [19, 55, 65].

Psychological flexibility will be measured with the 7-item Acceptance and Action Questionnaire-II (AAQ-II; [8]). Items will be scored on a 7-point scale ranging from never true (1) to always true (7). Sum scores will be calculated (7–49), with higher scores being indicative of larger psychological inflexibility levels. Psychometric properties were found to be good [8].

Positive coping skills will be measured with the subscales Acceptance, Positive refocusing, Positive reappraisal, and Putting into perspective of the Cognitive Emotion Regulation Questionnaire (CERQ; [35]). A 5-point scale ranging from (almost) never (1) to (almost) always (5). Sum scores will be calculated (4–20), with higher scores being indicative of a larger frequency of cognitive strategy use. Psychometric properties were found to be good [29, 60].

Mindfulness will be measured with the 15-item Mindfulness Attention Awareness Scale (MAAS; [11]). Items will be scored on a 6-point scale ranging from almost always (1) to almost never (6). Mean scores will be calculated, with higher scores being indicative of higher levels of mindfulness. Psychometric properties were evaluated and found to be good (e.g., [23]).

#### Characteristics of parents and children

Parental characteristics measured at screening include age and mental health support at the moment (i.e., whether parent self is in treatment for mental health issues) to determine eligibility. Parental characteristics measured at baseline (T0) include gender, age, education level, nationality, daily activities (e.g., employment), living situation (i.e., with/without partner, with one child with a NDD, with multiple children with a NDD, with one/more children with a NDD and one/more without a NDD), and marital status.

Characteristics about the child/children with a NDD include age, sex, diagnosis/diagnoses (i.e., the actual diagnosis, suspected of/formal diagnosis), language level (i.e., verbal, non-verbal, delay in language development), level of support required at school and in daily activities, sleeping issues (i.e., how well did the child sleep?), mental health support of the child(ren) (i.e., in the past or currently in treatment, use of medication). In case parents have multiple children with a NDD, they can respond for all children separately. To determine eligibility, the age and diagnosis are included in the screening, while the remaining characteristics are included in the baseline (T0) questionnaire.

#### Satisfaction with the intervention

Satisfaction with Adappt will be measured with the 8-item Client Satisfaction Questionnaire (CSQ-8; [4]). Satisfaction with the intervention will be measured in terms of quality of the intervention, fulfillment of needs and expectations, recommendation to other parents in a similar situation, helpfulness of the self-help app, satisfaction with the self-help app in general, and amount of support offered by the self-help app. Items will be scored on a 4-point scale, and a sum score (8–32) will be calculated with higher scores being indicative of a larger satisfaction with the self-help app. An excellent internal consistency was found by Kloos et al. [48].

Additional evaluative questions will be asked to get more insight in the evaluation of the app: (1) lessons learned from the self-help app, (2) themes that were perceived to be effective, (3) adherence as indicated by the number of days per week used, and minutes used per time, (4) functionalities (e.g., texts, videos, exercises, notifications/reminders, quotes, seeing progress, mindfulness exercises, traveling diary), and (5) content of the intervention (i.e., language used, amount of text, design, ease of use, and number of themes).

#### Logdata

Pseudonymized logdata will be collected automatically by the application. This logdata consists of information whether an exercise have been completed.

#### Dropout and retention

Dropouts will be asked to give feedback on the reason of withdrawal if they want to by asking “why did you decide to stop using the app?”. If a participant mentions problems with the app to be the reason, these will be communicated with the app developer and if fixed, the user will be contacted about this. Furthermore, to promote completion of the questionnaires, two reminders will be sent after an assessment point to non-completers.

### Data handling, management, and storage

The Dutch Personal Data Protection Act will be followed to handle data with confidentiality. Data management will be done following the data management plan (DMP) created for the study via the DMP-tool of the University of Twente, which complies to the policy of national funders and the EU. Personal data (i.e., e-mail addresses) will be collected for the purpose of sending questionnaires via the Qualtrics survey tool, which is ISO 270001 certified [59]. Personal data will be saved separately from the questionnaire data and can be linked to questionnaire data by means of a unique ID-code. Personal data can be accessed by the principal investigator for the purpose of informing participants about the screening for eligibility data and sending the questionnaire invitations. The list with IDs connected to personal data will be stored safely and locked with a password. The coded data will be safely stored for 15 years at the IGS Datalab of the University of Twente, which is ISO 27001 and NEN 7510-certified [75]. The first, second, third, and last author (KT, CD, PK, ED) of this paper will have access to the final pseudonymized dataset of this trial. Afterwards, pseudonymized data will be archived for long-term storage at Areda, the archiving service of the University of Twente. Participants get access to the self-help app via a personal activation code, this makes the log-data linkable with the study data (i.e., results from questionnaires).

### Statistical methods

#### Sample size calculation

The required sample size was computed with RMASS2 software for two-group longitudinal designs with attrition [39]. Given a required power of 80% and a two-sided significance level of 5%, 106 participating parents will need to be recruited in each arm to detect a linear trend effect over the first three time points (a group by linear time interaction from T0 to T2) resulting in at least a moderate effect size difference between the groups (Cohen’s *d* = 0.5) at 4 months after baseline. So, at least 212 parents must be included in this RCT.

This sample size estimation is based on the assumption that the repeated measurements follow a first-order autoregressive (AR1) variance–covariance structure (starting at *ρ* = 0.5 between repeated measurements) and an assumed attrition of 10% between both the baseline (T0) and post-intervention (T1) and between the post-intervention (T1) and 4 month follow-up (T2) measurements. Given the relatively low number of involved countries (five) in the trial and the self-help nature of the intervention, no relevant clustering between participants at the country level is anticipated in the sample size calculation.

#### Statistical analysis

Data of participants that provided incomplete baseline data will be removed from the dataset.

The study and data will be reported conform the Consolidated Standards of Reporting Trials (CONSORT) statement [62]. Data will be primarily analyzed based on intention-to-treat with linear mixed models (LMMs), since LMM fits the nested, repeated measures structure of this study, and can inherently deal with missing data on the outcome variable. The effectiveness of the app on all continuous primary and secondary outcomes, and potential working mechanisms between baseline (T0) to the first follow-up (T2) will be analyzed using LMMs with time, group and time-by-group interactions at fixed effects and with baseline scores as covariate [74].

Three additional within-group analyses will be performed by a LMM with time as the main effect to determine if intervention effects remained between T2 and T3 (in the intervention condition) and if there are improvements in the control condition after using the intervention at T3 (between T2 and T3). Between and within-group Cohen’s *d* effect sizes with 95% confidence intervals will calculated to estimate (potential) benefits of the intervention based on estimated marginal means and standard errors (from the LMMs). User satisfaction with the self-help app will be descriptively analyzed.

Single (one explanatory variable) and multiple (all five explanatory variables) mediation analysis will be performed to assess whether the explanatory variables mediated the effects of the intervention on the ability to adapt, mental well-being, perceived stress, depressive symptoms, and anxiety symptoms.

### Ethics

Before the start of the study, a non-WMO declaration of the Medical Ethical Committee (METC) Oost Nederland and ethical approval of the ethics committee of the University of Twente (approved at January 10, 2024) were obtained. Amendments of the protocol will be re-evaluated by the METC Oost Nederland, the ethical board of the University of Twente, and communicated with the trial register. The study will be conducted in accordance with the principles of the Declaration of Helsinki (64th WMA General Assembly, Fortaleza, Brazil, October 2013) and with the Good Clinical Practice guidelines. Individual clinical trial participant-level data (IPD) will not be shared.

### Risks and audit

Participants will be informed on the study and give informed consent before the start of the trial. After the screening, participants with major mental health issues will be advised to go to their general practitioner. Based on previous studies of comparable applications for other target groups [48, 74], no risks of participation are foreseen. As this study is declared to be non-medical (non-WMO), audits and a data monitoring committee are not planned.

## Results

Data collection is expected to be completed by the end of 2025.

### Publication policy

Results (on group level) of this study will be published in at least one international peer-reviewed journal. Results will be reported in a final report for the subsiding party (the European Union). No specific arrangements are made between the sponsor and the investigator concerning the public disclosure and publication of the research data. Participants can receive a summary of results (on group level) of the study upon request.

## Discussion

This protocol describes a study on the short- and long-term effectiveness of a self-help app based on positive psychology and mindfulness to support parents of children with a NDD in enhancing the sense of ability to adapt, and mental health. Furthermore, mediation analyses will be performed to find working mechanisms appearing to contribute to the effectiveness of the self-help app. Participants will be recruited in five countries, i.e., France, Germany, Israel, Switzerland, and The Netherlands; however, there are no limitations to the country participants live in, except for being able to speak one of the offered languages (French, German, Hebrew, or Dutch). The study will compare the intervention with a delayed waitlist control condition. The described study will contribute to knowledge on the effectiveness of support for parents of children with a NDD and give insight into working mechanisms that contribute to this effectiveness.

Several strengths are expected for the intervention and study. Firstly, this intervention has been developed in cocreation with the target group to meet the needs and preferences of the target group. Secondly, the exercises in the app are evidence based, increasing the chance of effectiveness of the intervention. Thirdly, the intervention involves a self-help app, having the advantage of being cost-effective, usable at any time the user prefers to, and easily scalable. Fourthly, this study adds to existing research by including mediation analysis to find working mechanisms. Fifthly, this is one of the first RCTs with a longer follow-up (6 months after the intervention).

Several limitations are also expected in advance. Firstly, it is expected that majority of participants will be mothers, limiting the generalizability of conclusions to fathers. This is expected since the recruitment of participants for the development phase has shown that recruiting fathers is much harder than recruiting mothers. This is also in line with research on the effectiveness of positive psychology interventions across different populations, since the bibliometric analysis of Hendriks et al. [41] showed that almost three-quarters of participants was female in 180 studies. Furthermore, mothers are perceived to be the primary caregiver of children with a NDD [2, 50]. Secondly, study dropout and intervention non-adherence can result in bias [28]. Post hoc analysis will be performed to compare baseline characteristics of study dropouts at post-intervention, 3-month follow-up, and 6-month follow-up.

## Trial status

*Protocol version:* version 12, March 25, 2024.

*Recruitment status*: pending

*Recruitment start*: September 2024.

*Recruitment completed*: expected in January 2025.

### Supplementary Information

Supplementary Material 1.

## Data Availability

Data will be made available upon request.
